# Supplementary material for: Evaluation of the Structural Modification of Ibuprofen on the Penetration Release of Ibuprofen from a Drug-in-Adhesive Matrix Type Transdermal Patch
Source: Int J Mol Sci. 2022 Jul 13;23(14):7752. doi: 10.3390/ijms23147752 (PMC9320482; doi:10.3390/ijms23147752)
Supplement: Supplementary file 1 [file ijms-23-07752-s001.zip › ijms-1807129-supplementary.pdf]

Supplementary data

Evaluation of structural modification of ibuprofen on the penetration release of ibuprofen from drug-in-adhesive matrix type transdermal patch

Paula Ossowicz-Rupniewska[a*], Paulina Bednarczyk[a], Małgorzata Nowak[a], Anna Nowak[b], Wiktoria Duchnik[b], Łukasz Kucharski[b], Joanna Klebeko[a], Ewelina Świątek[a], Karolina Bilska[a], Zbigniew Czech[a]

[a] West Pomeranian University of Technology in Szczecin, Faculty of Chemical Technology and Engineering, Department of Chemical Organic Technology and Polymeric Materials, Piastów Avenue 42, 71-065 Szczecin, Poland

[b] Pomeranian Medical University in Szczecin, Department of Cosmetic and Pharmaceutical

*Corresponding author: Paula Ossowicz-Rupniewska, e-mail: possowicz@zut.edu.pl; tel: +48914494801

Number of pages: 15
Number of Figures: 26
Number of Tables: 1

Table of Contents

1. Microspectroscopy analysis of the transdermal patch S2-S6
2. The TG curves of obtained patches S7-S13
3. The DSC curves of obtained patches S13-S19
4. The Mann-Whitney's test estimated significant differences in the cumulative mass between all analyzed compounds, taking all time points during the entire 24 h permeation. S20

Microspectroscopy analysis of the transdermal patch

A

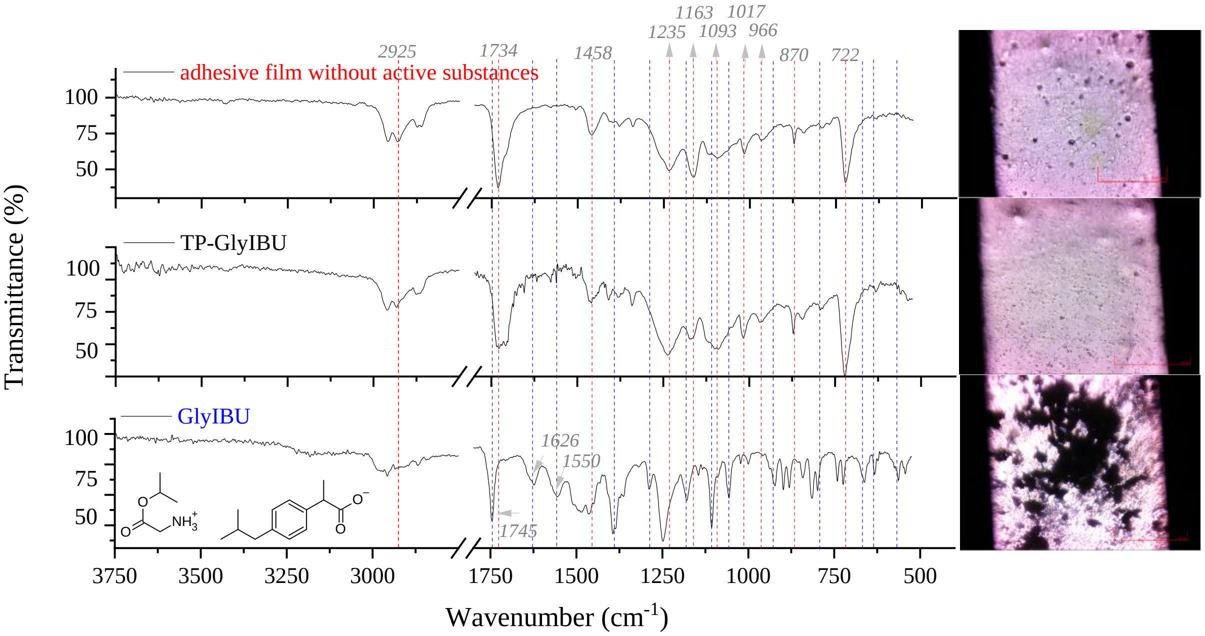

B

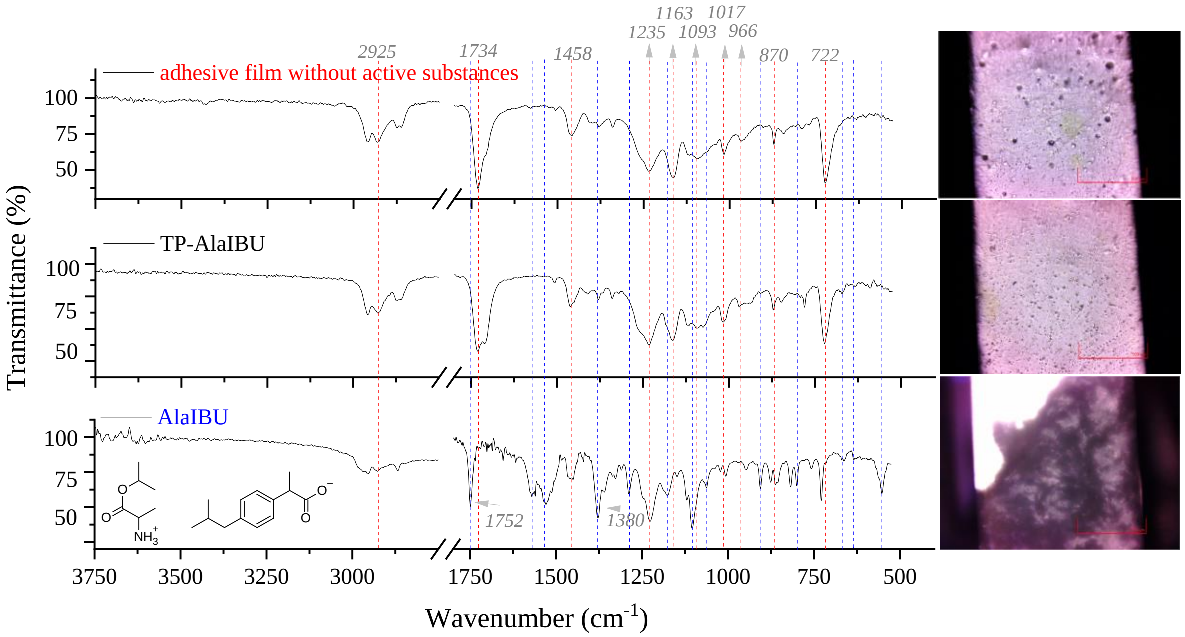

C

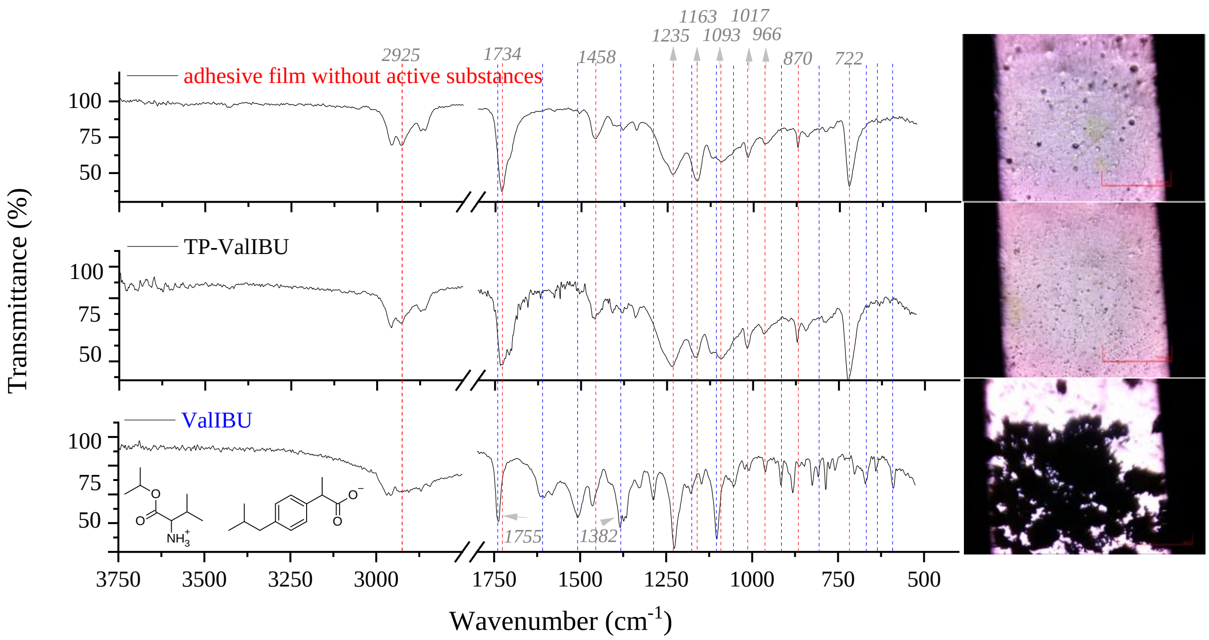

adhesive film without active substances
TP-ValIBU
ValIBU
2925
1734
1458
1235
1163
1093
1017
966
870
722
1755
1382
Transmittance (%)
Wavenumber (cm-1)

D

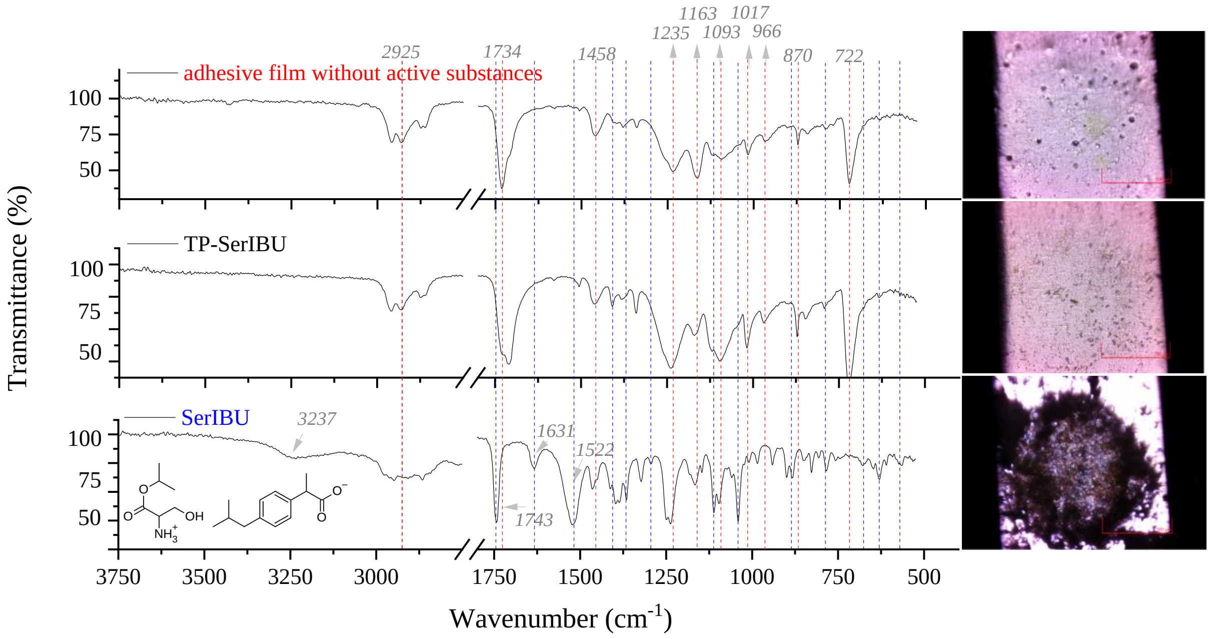

adhesive film without active substances
TP-SerIBU
SerIBU
3237
1631
1522
1743
2925
1734
1458
1235
1163
1093
1017
966
870
722
Transmittance (%)
Wavenumber (cm-1)

E

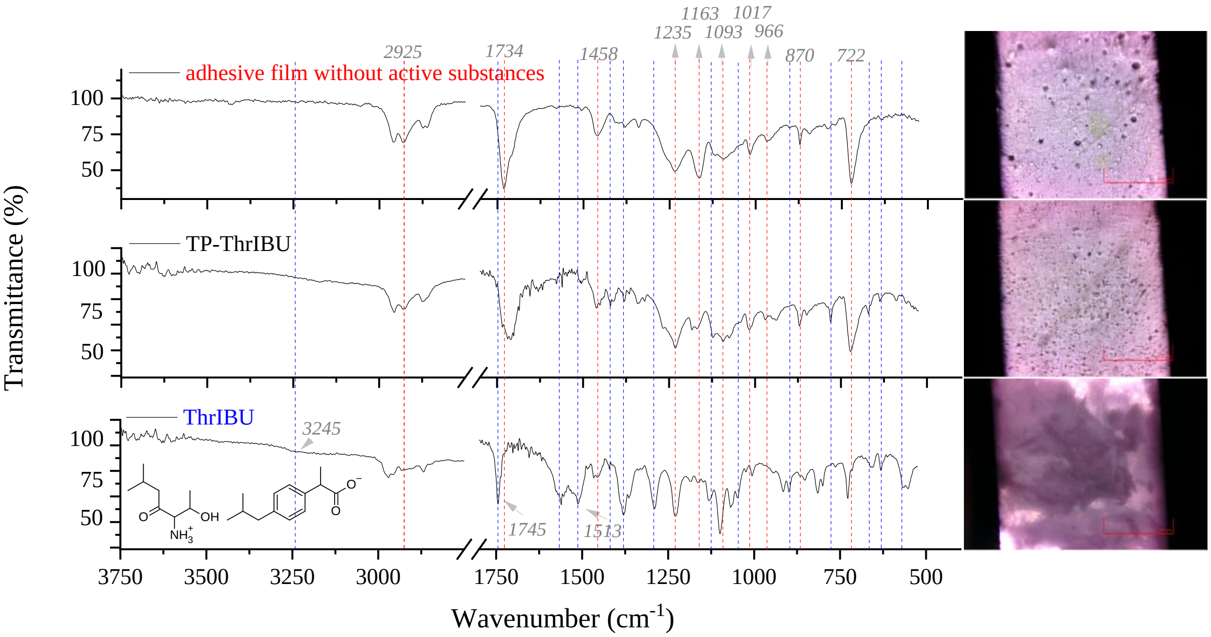
adhesive film without active substances
TP-ThrIBU
ThrIBU
Transmittance (%)
Wavenumber (cm-1)
2925
1734
1458
1235
1163
1093
1017
966
870
722
3245
1745
1513
100
75
50
3750
3500
3250
3000
1750
1500
1250
1000
750
500

F

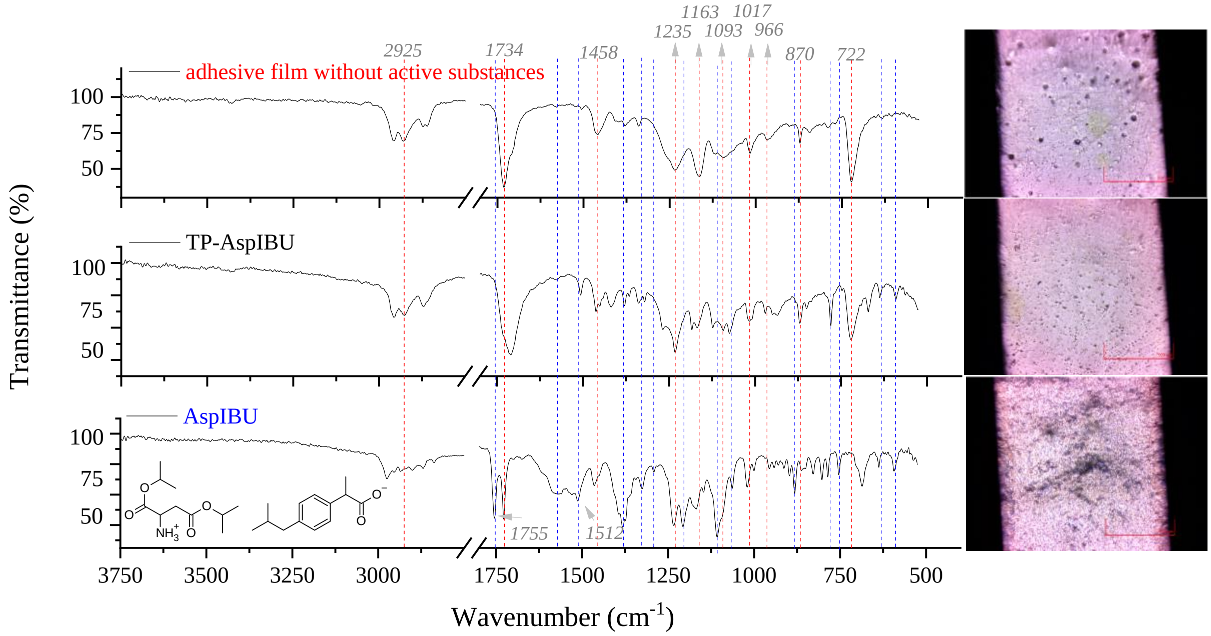
adhesive film without active substances
TP-AspIBU
AspIBU
Transmittance (%)
Wavenumber (cm-1)
2925
1734
1458
1235
1163
1093
1017
966
870
722
1755
1512
100
75
50
3750
3500
3250
3000
1750
1500
1250
1000
750
500

G

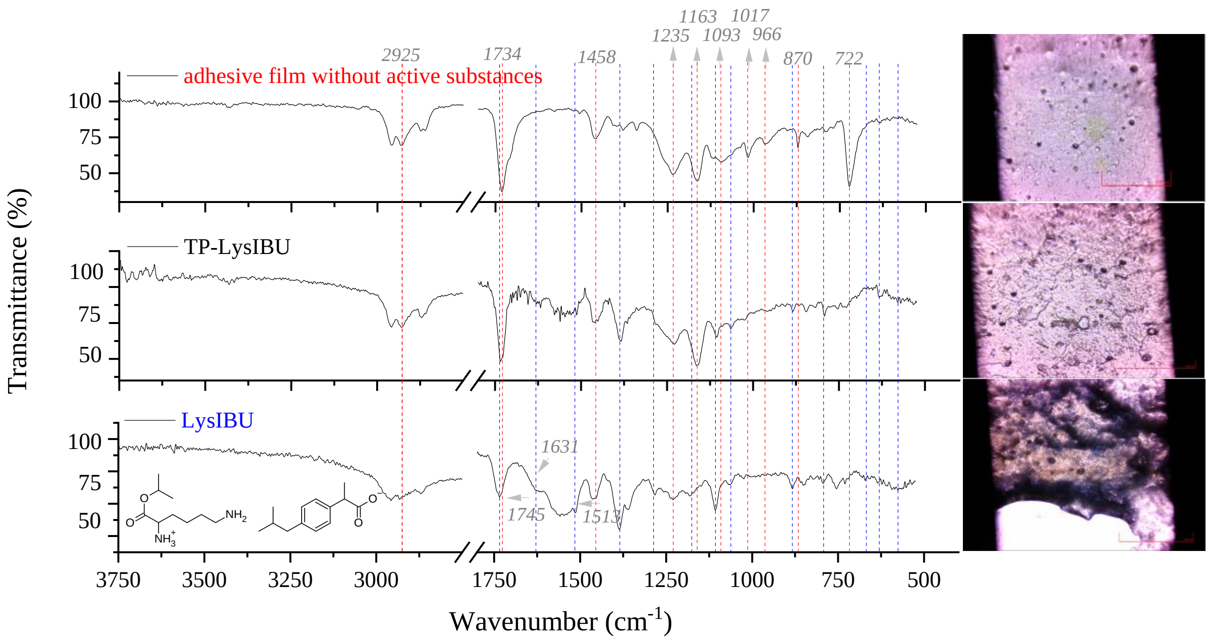
2925
1734
1458
1235
1163
1093
1017
966
870
722
adhesive film without active substances
TP-LysIBU
LysIBU
1631
1745
1513
Transmittance (%)
Wavenumber (cm-1)

H

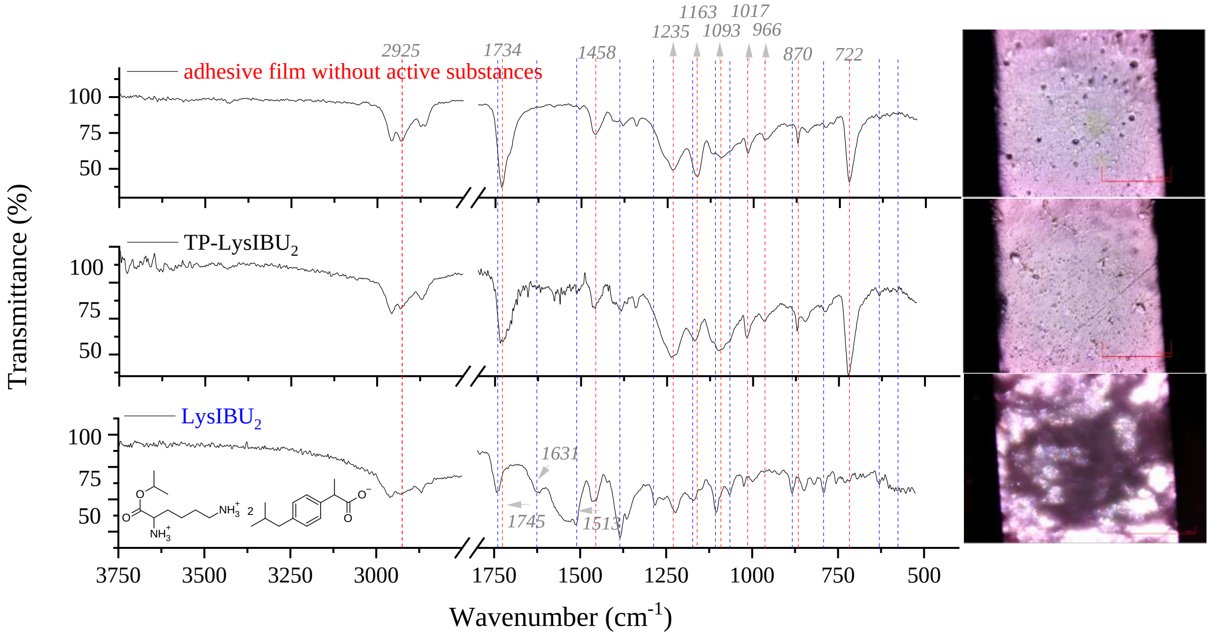
2925
1734
1458
1235
1163
1093
1017
966
870
722
adhesive film without active substances
TP-LysIBU2
LysIBU2
1631
1745
1513
Transmittance (%)
Wavenumber (cm-1)

I

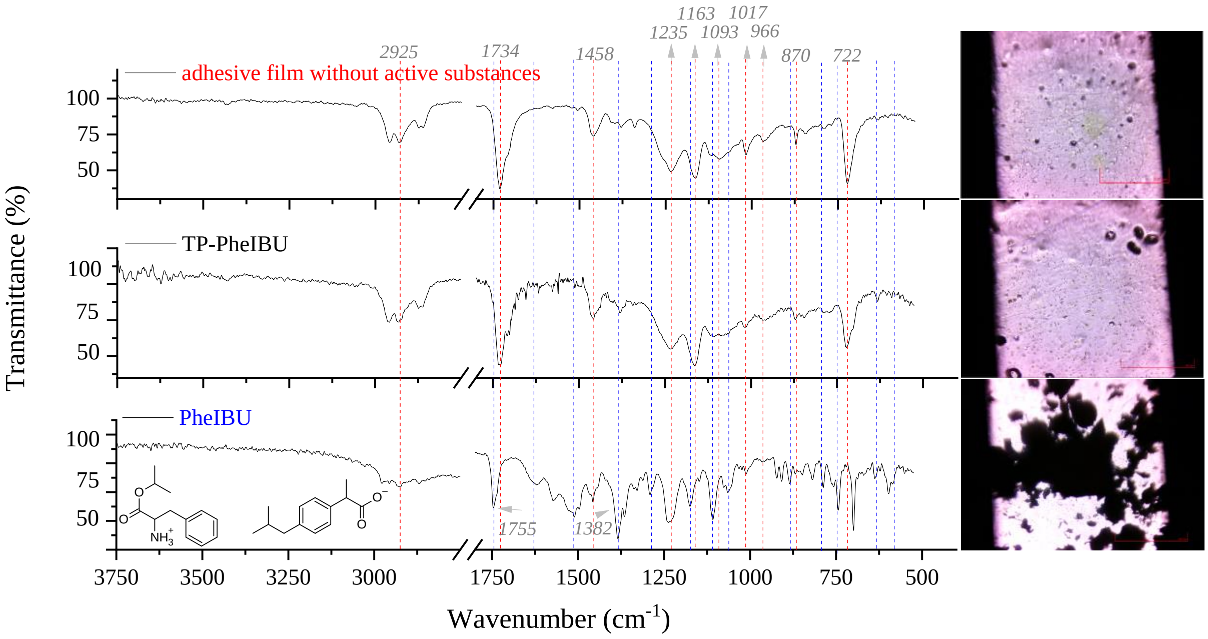

J

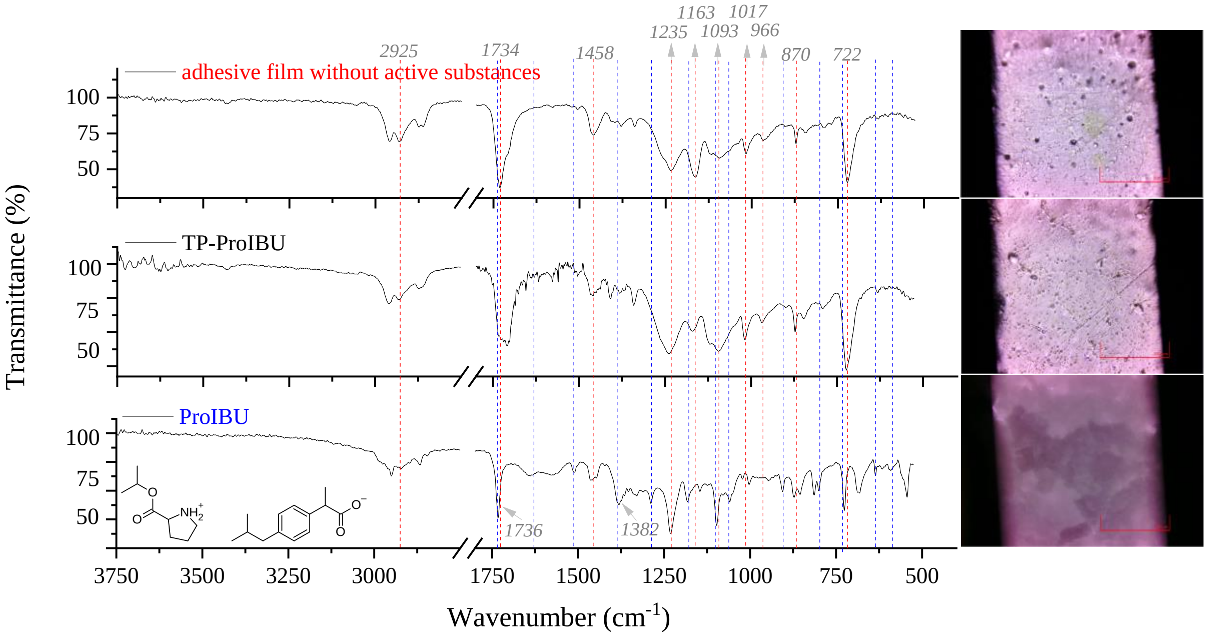

Figure S1. Microspectroscopy analysis of the transdermal patch (TP) – (A) with GlyIBU; (B) with AlaIBU; (C) with ValIBU; (D) with SerIBU; (E) with ThrIBU; (F) with AspIBU; (G) with LysIBU; (H) with $LysIBU_2$; (I) with PheIBU; (J) with ProIBU.

The TG curves of obtained patches

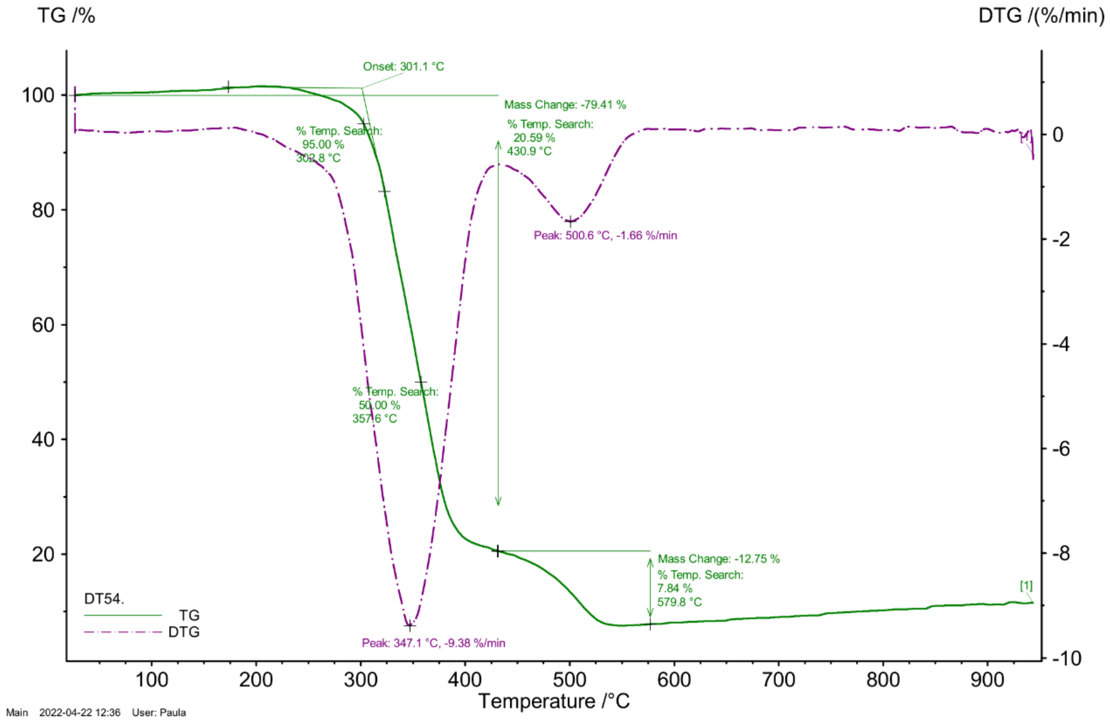

Figure S2. The TG and DTG curves of DT54 patch.

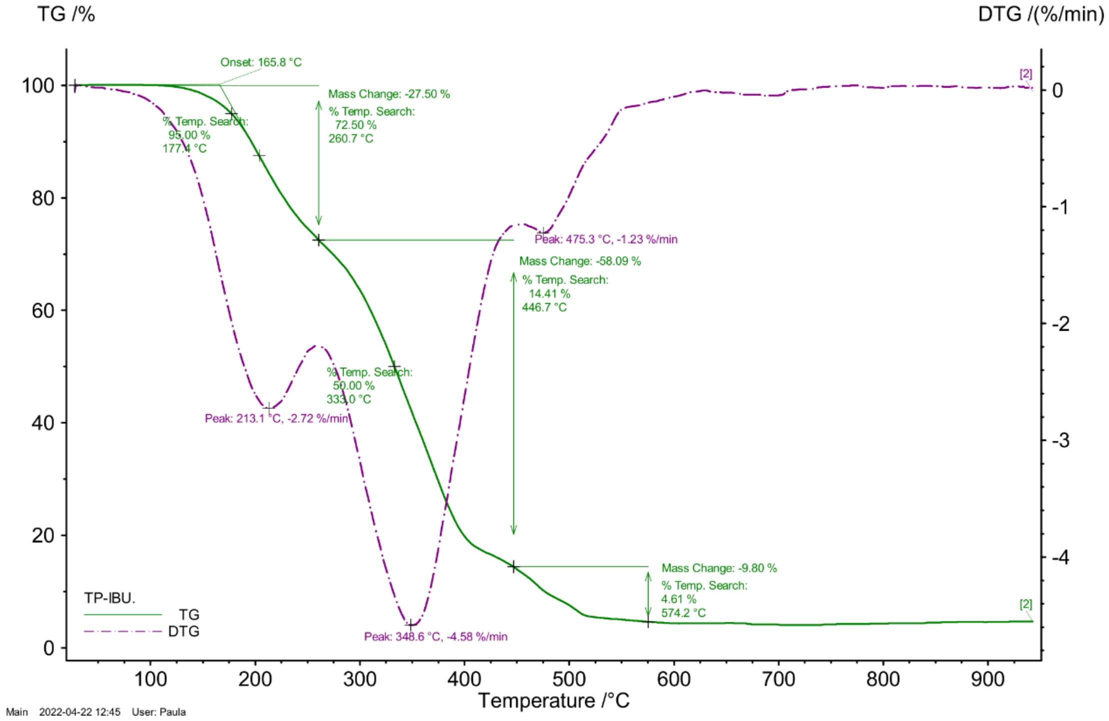

Figure S3. The TG and DTG curves of TP-IBU patch.

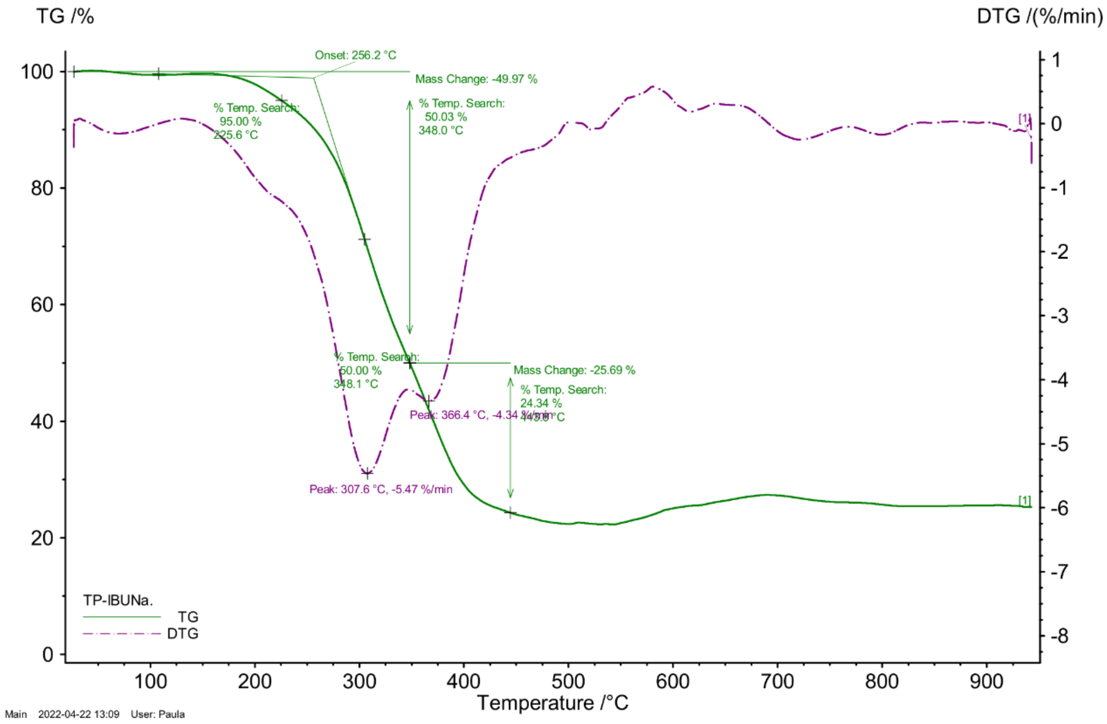

Figure S4. The TG and DTG curves of TP-IBUNa patch.

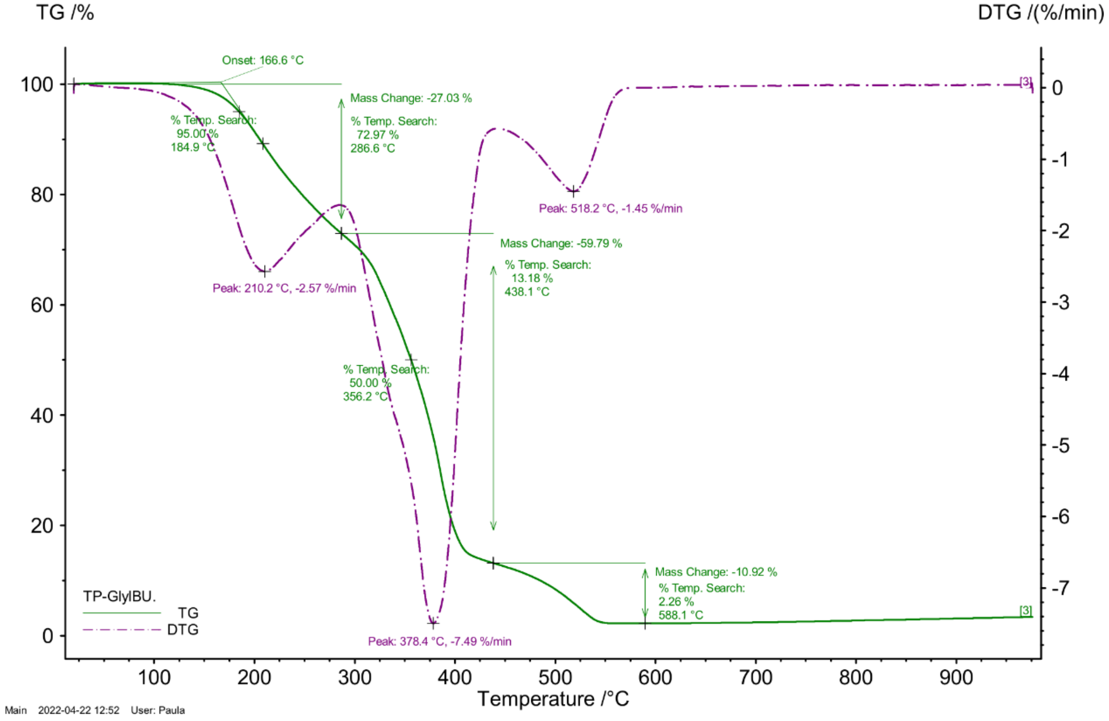

Figure S5. The TG and DTG curves of TP-GlyIBU patch.

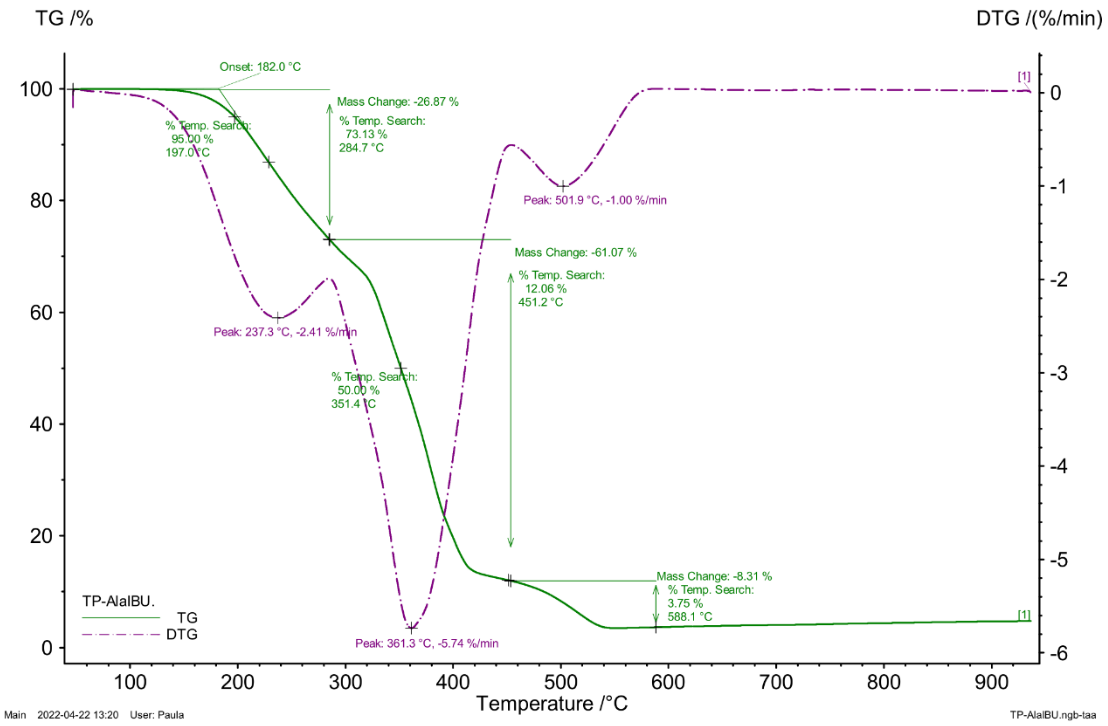

Figure S6. The TG and DTG curves of TP-AlaIBU patch.

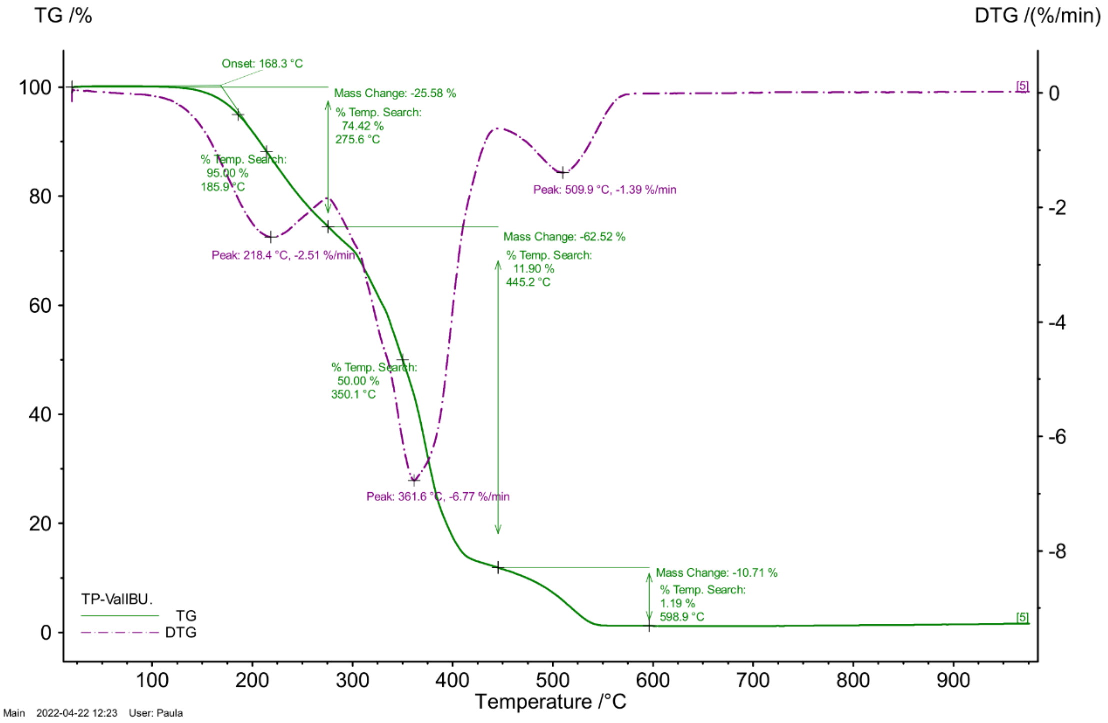

Figure S7. The TG and DTG curves of TP-ValIBU patch.

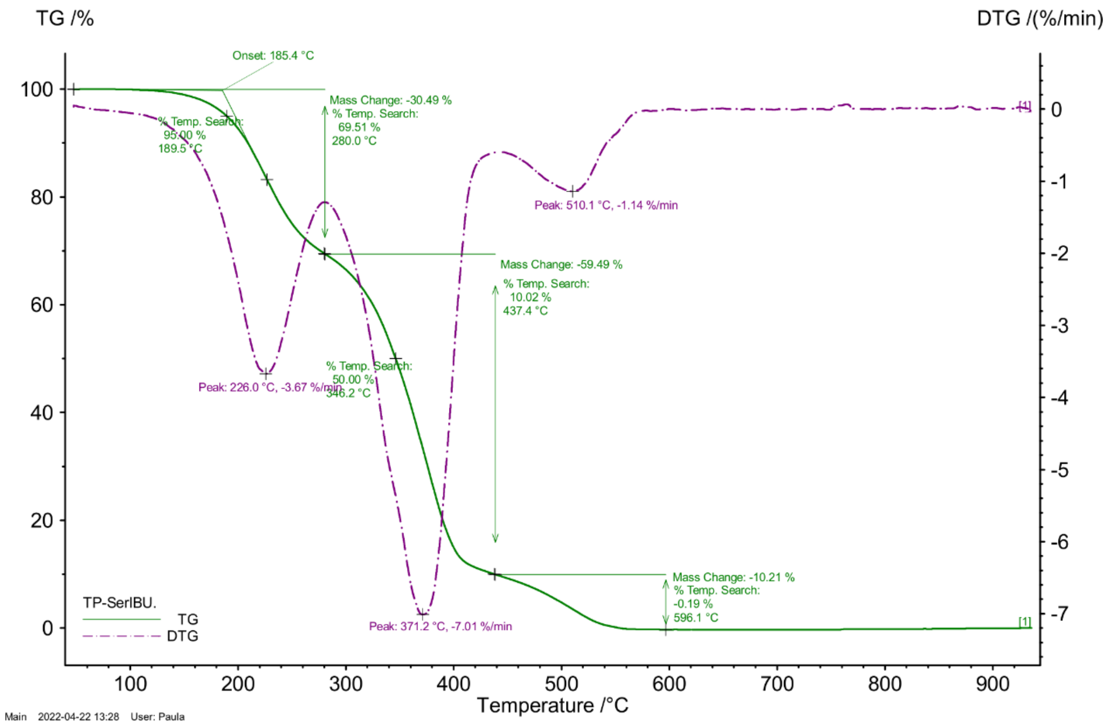

Figure S8. The TG and DTG curves of TP-SerIBU patch.

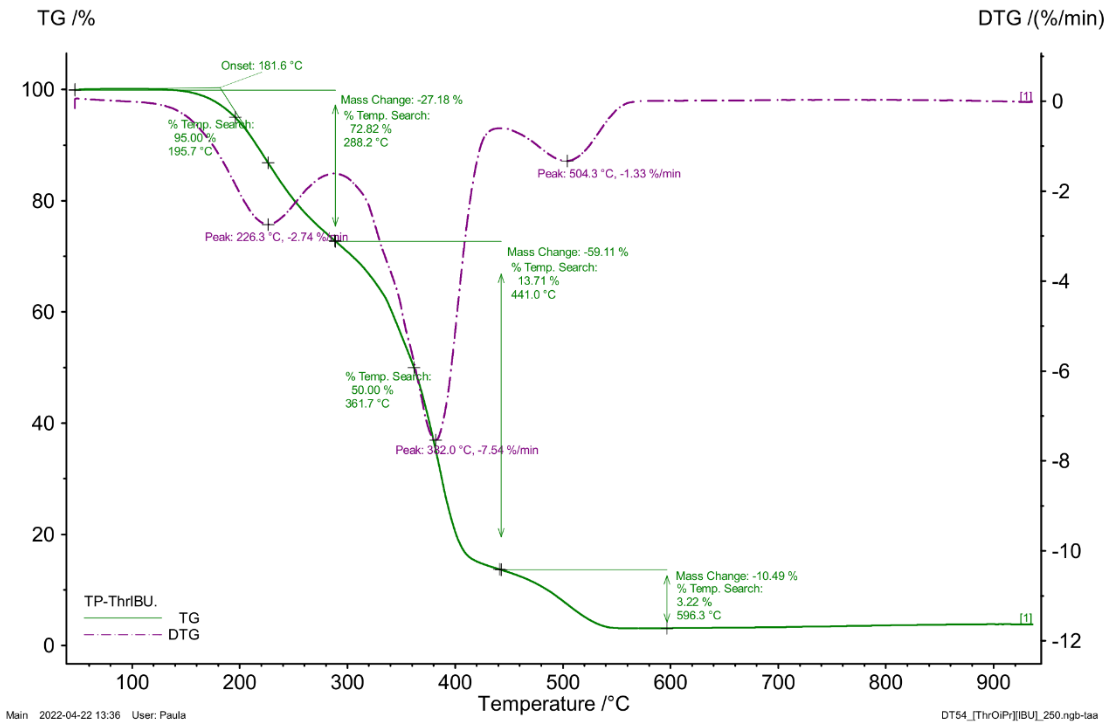

Figure S9. The TG and DTG curves of TP-ThrIBU patch.

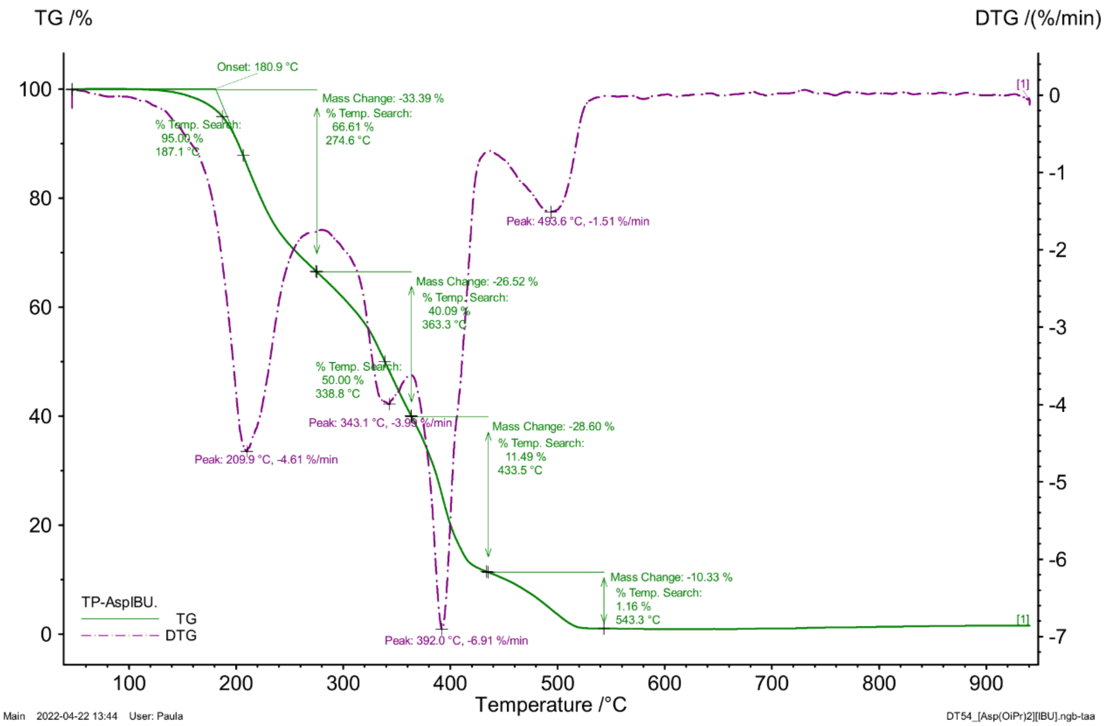

Figure S10. The TG and DTG curves of TP-AspIBU patch.

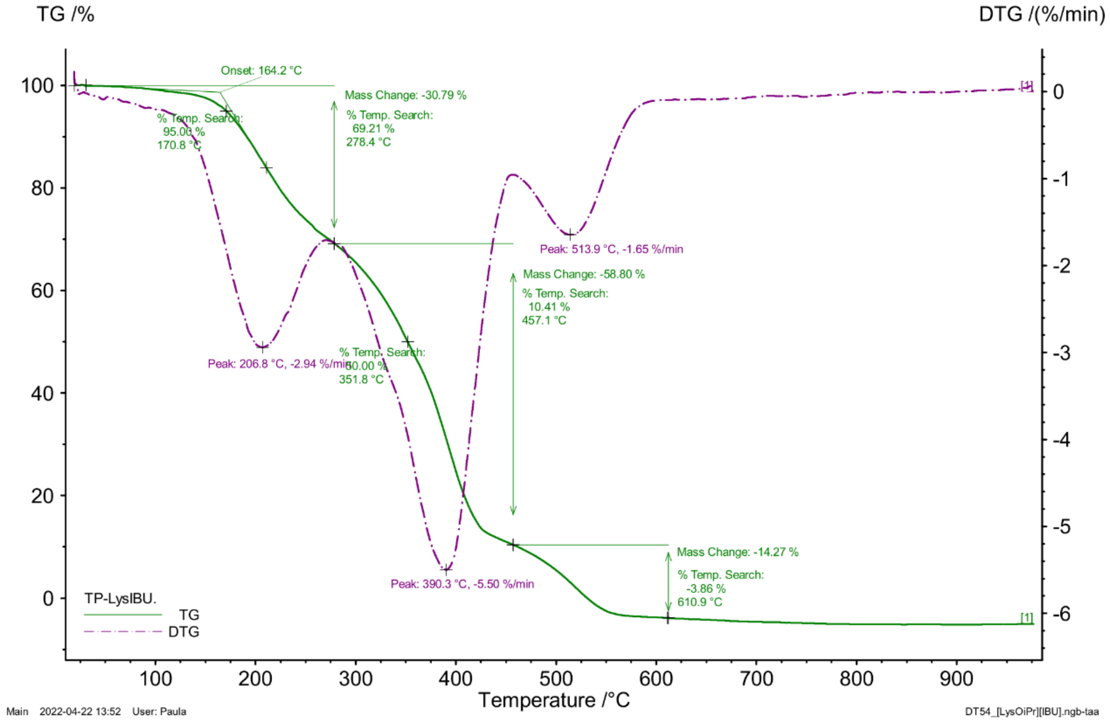

Figure S11. The TG and DTG curves of TP-LysIBU patch.

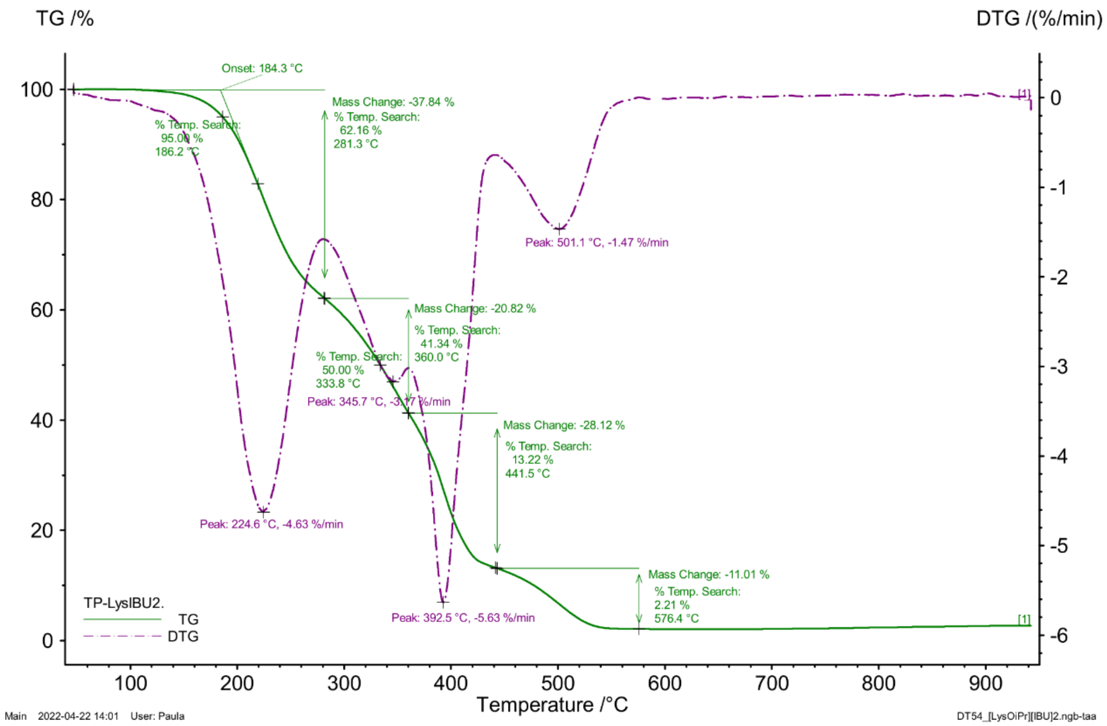

Figure S12. The TG and DTG curves of TP-LysIBU$_2$ patch.

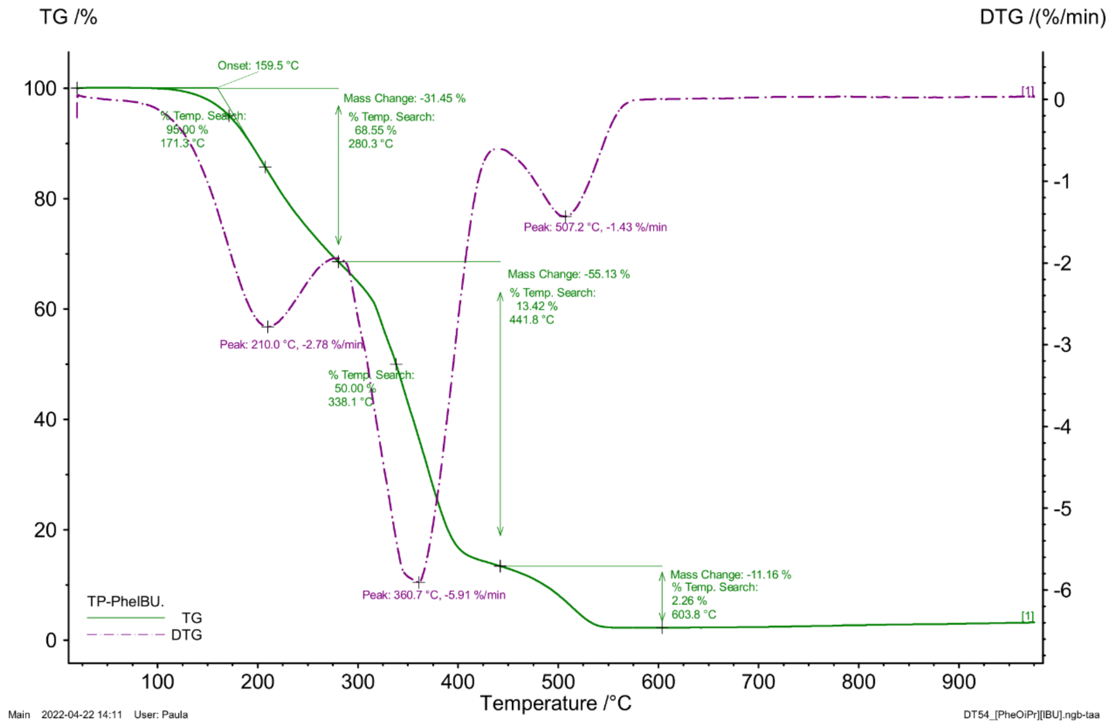

Figure S13. The TG and DTG curves of TP-PheIBU patch.

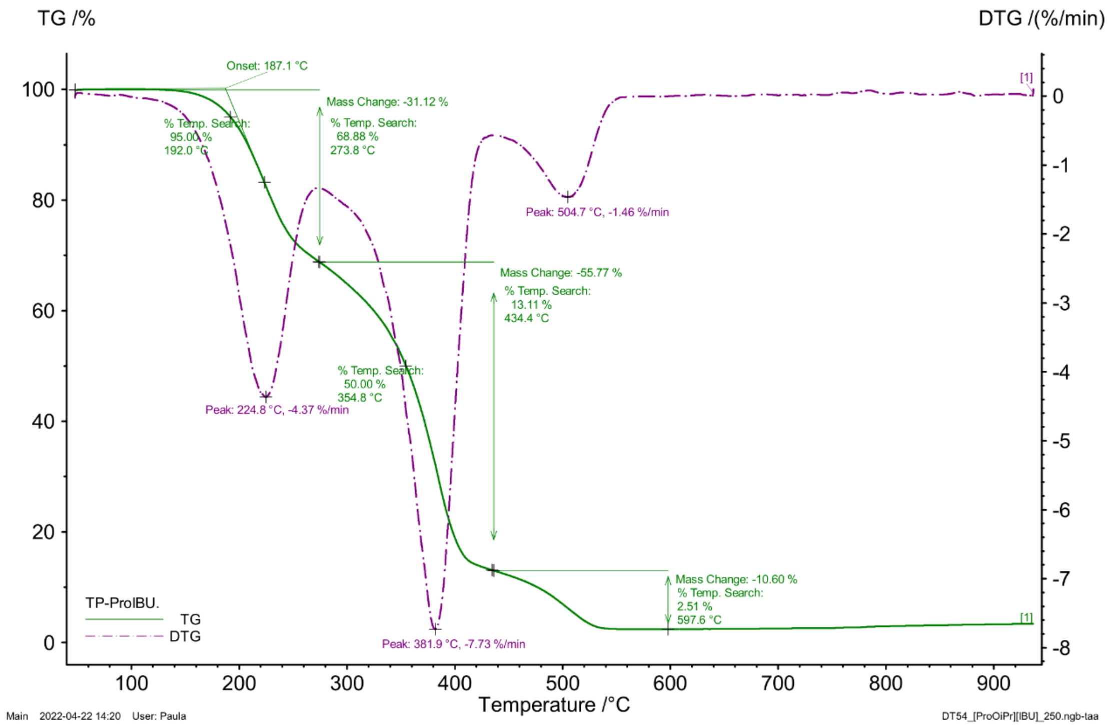

Figure S14. The TG and DTG curves of TP-ProIBU patch.

The DSC curves of obtained patches

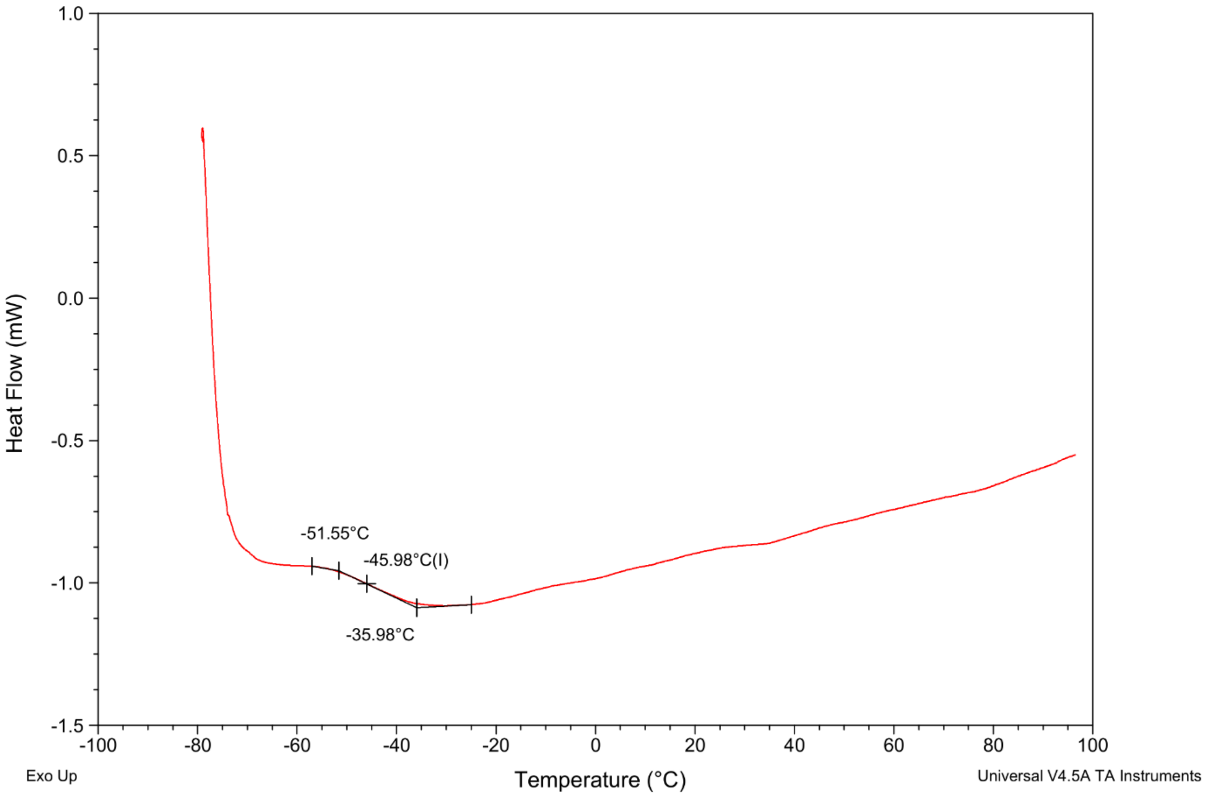

Figure S15. The DSC curve of DT54 patch.

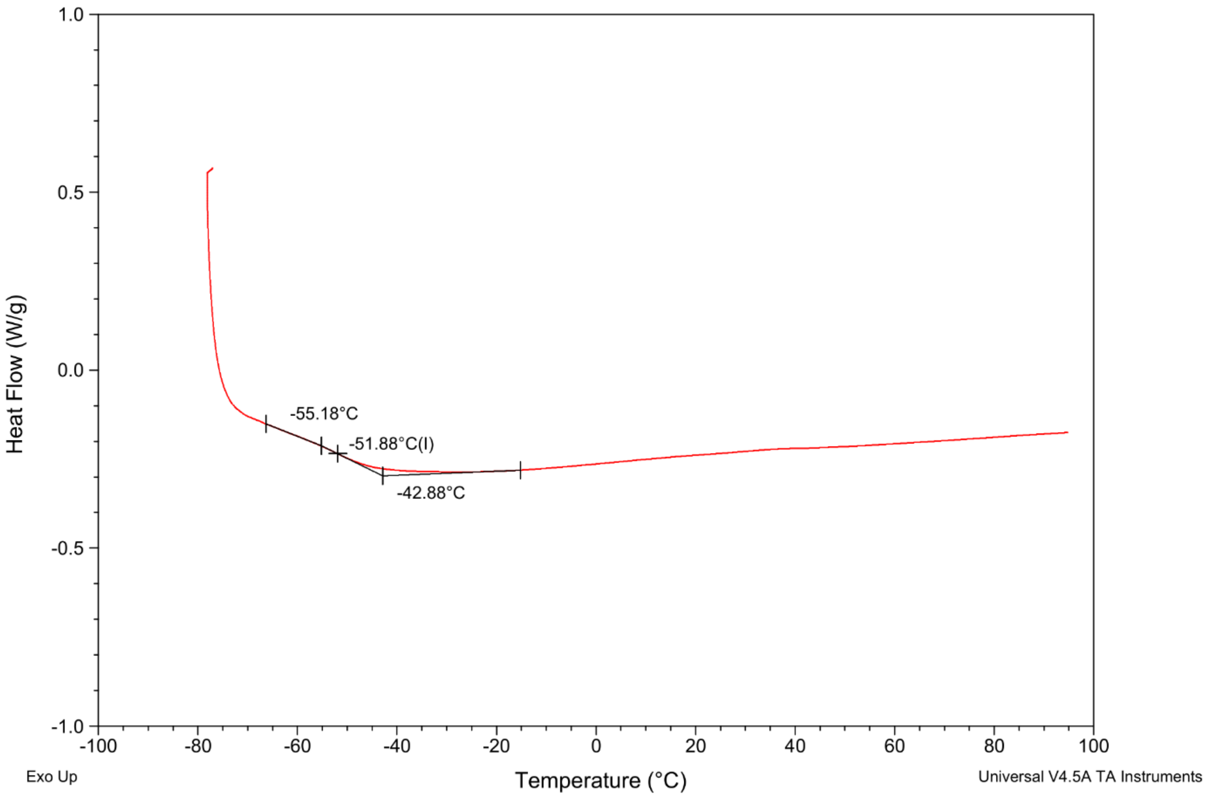

Figure S16. The DSC curve of TP-IBU patch.

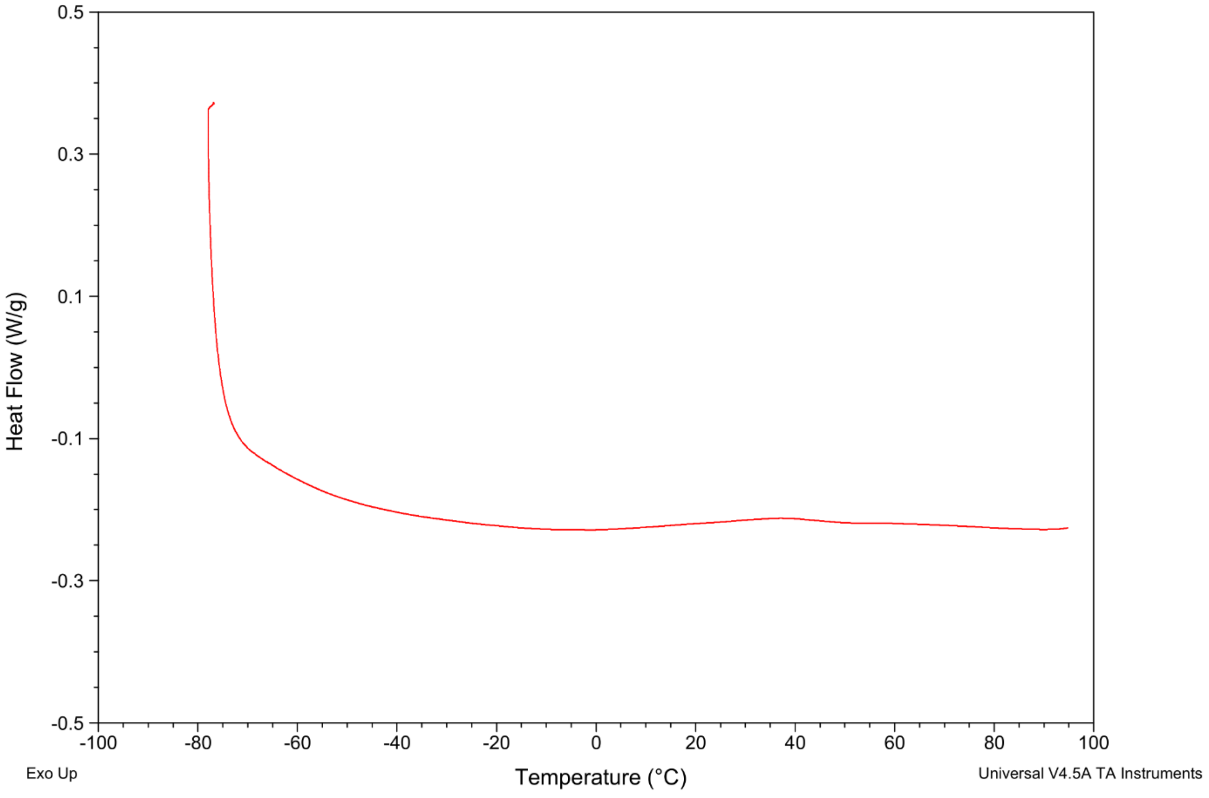

Figure S17. The DSC curve of TP-IBUNa patch.

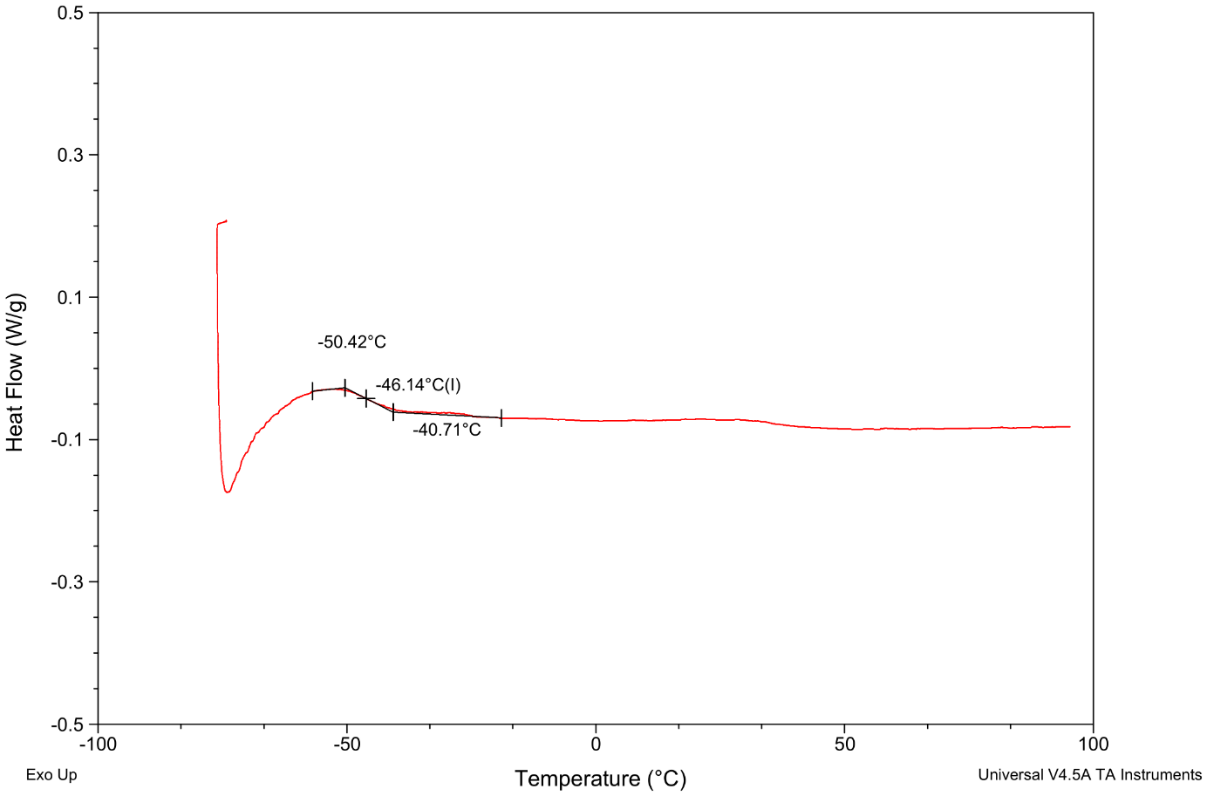

Figure S18. The DSC curve of TP-GlyIBU patch.

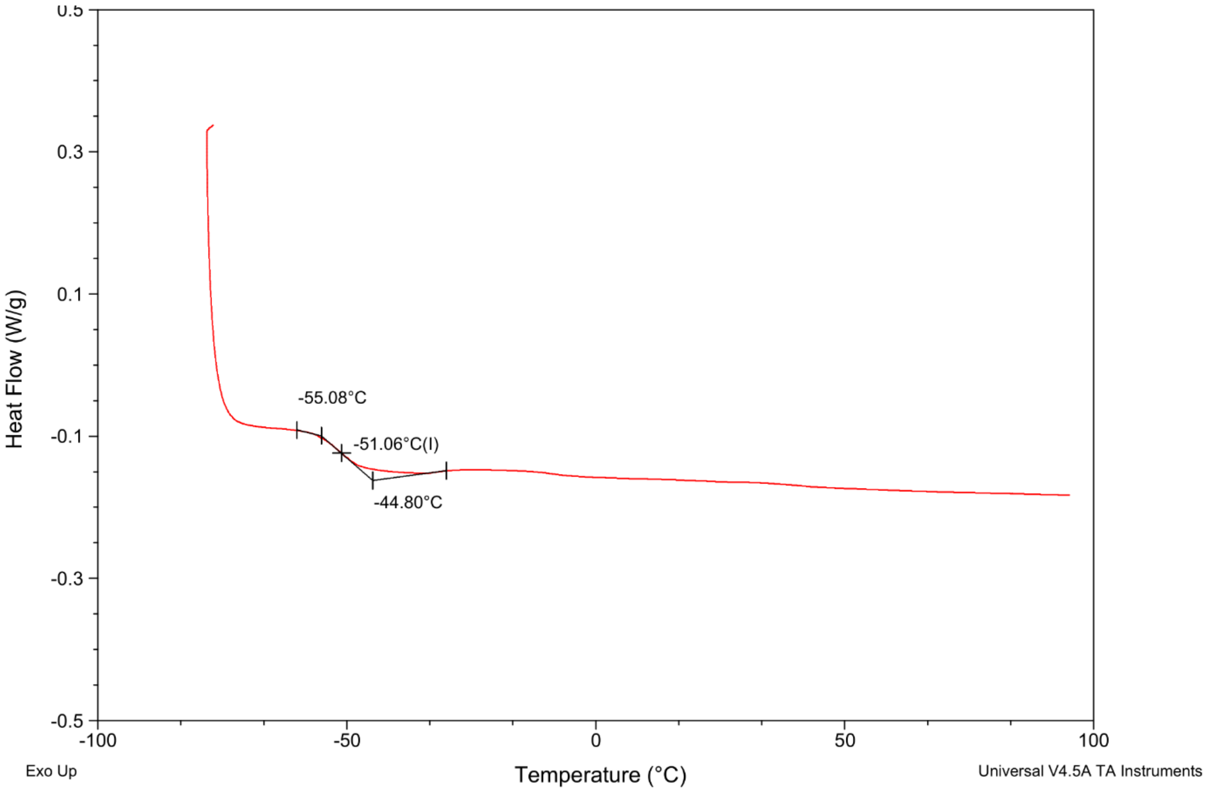

Figure S19. The DSC curve of TP-AlaIBU patch.

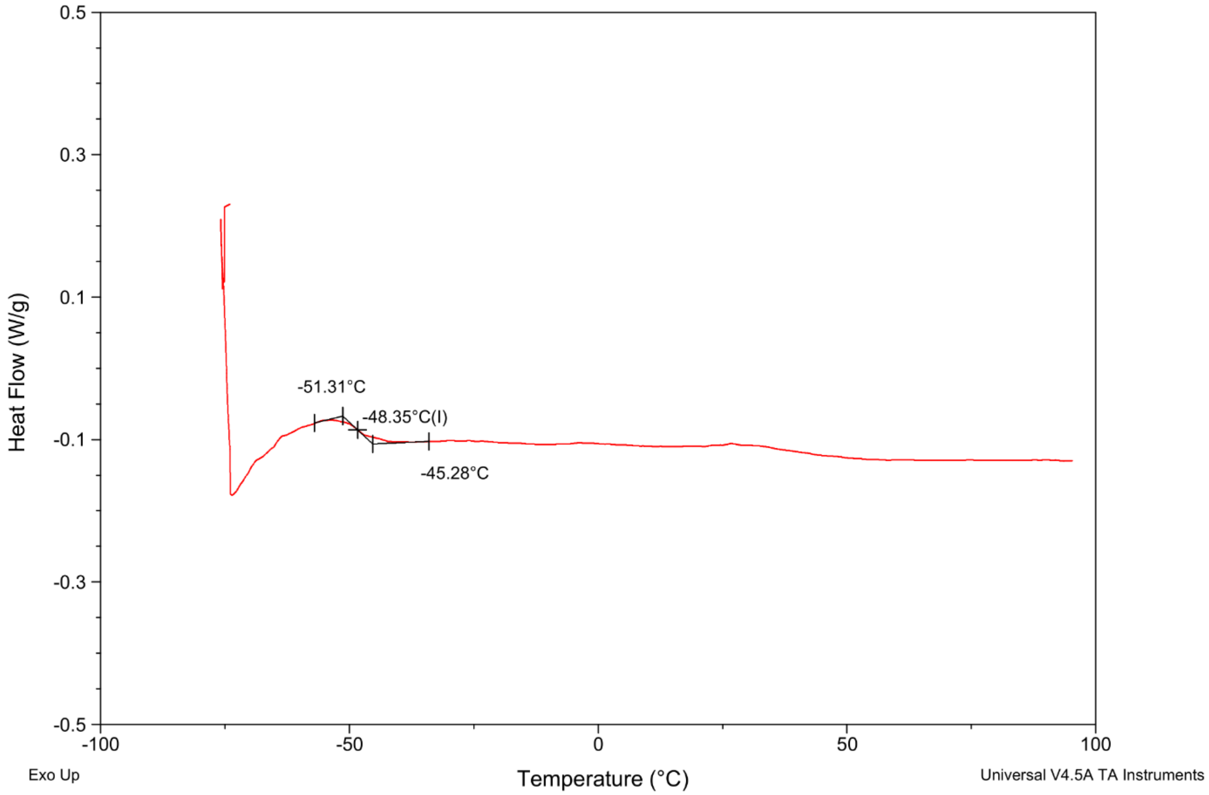

Figure S20. The DSC curve of TP-ValIBU patch.

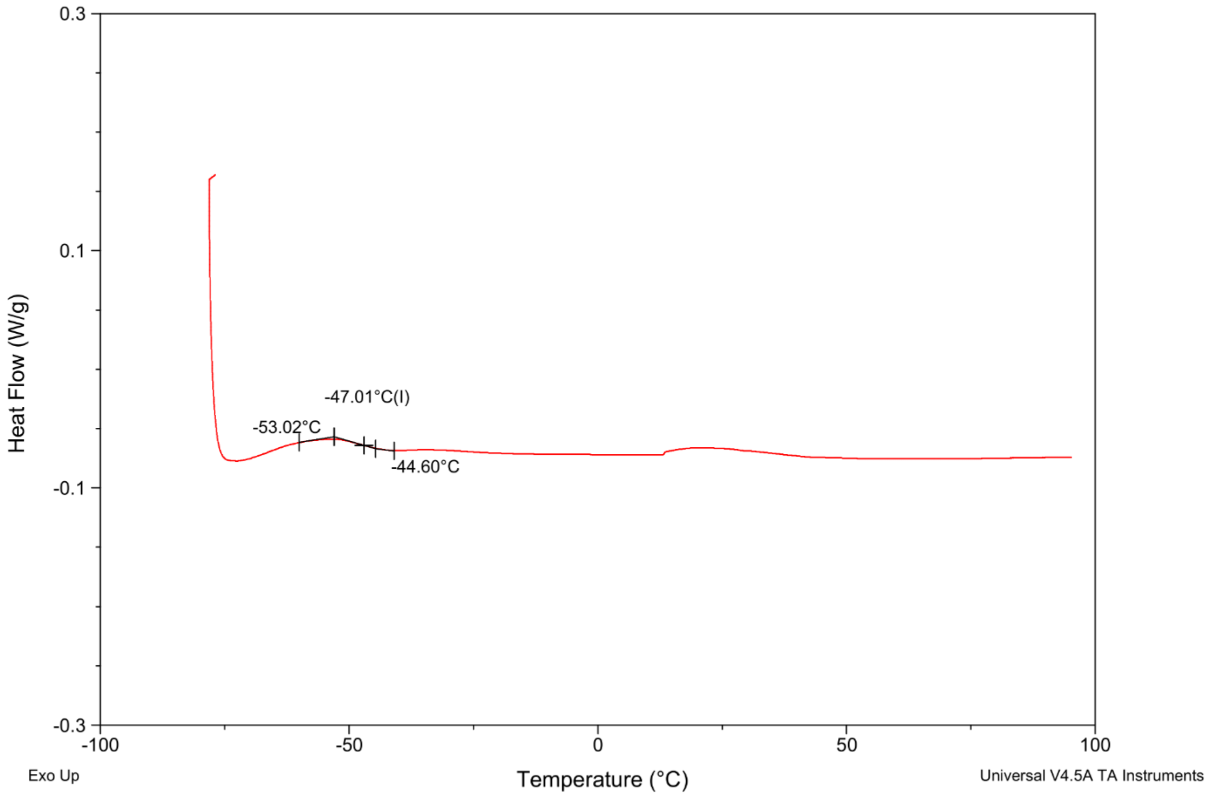

Figure S21. The DSC curve of TP-SerIBU patch.

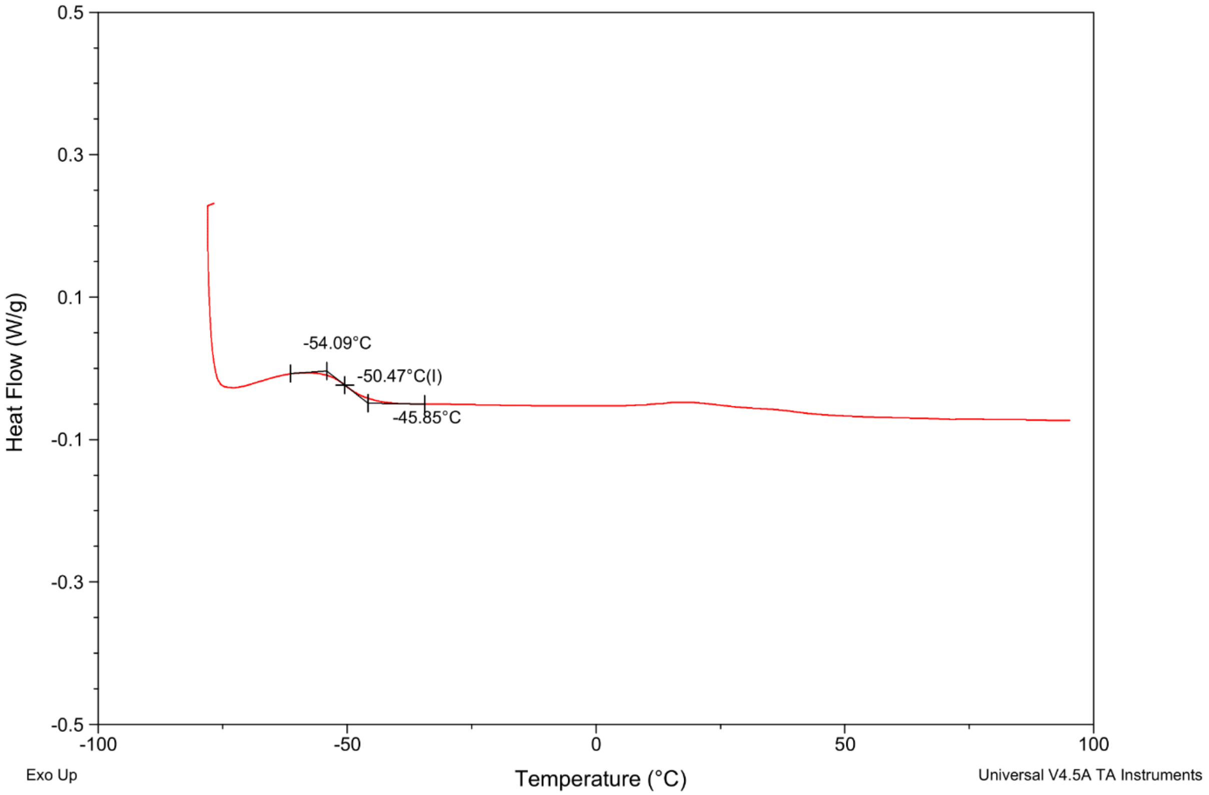

Figure S22. The DSC curve of TP-ThrIBU patch.

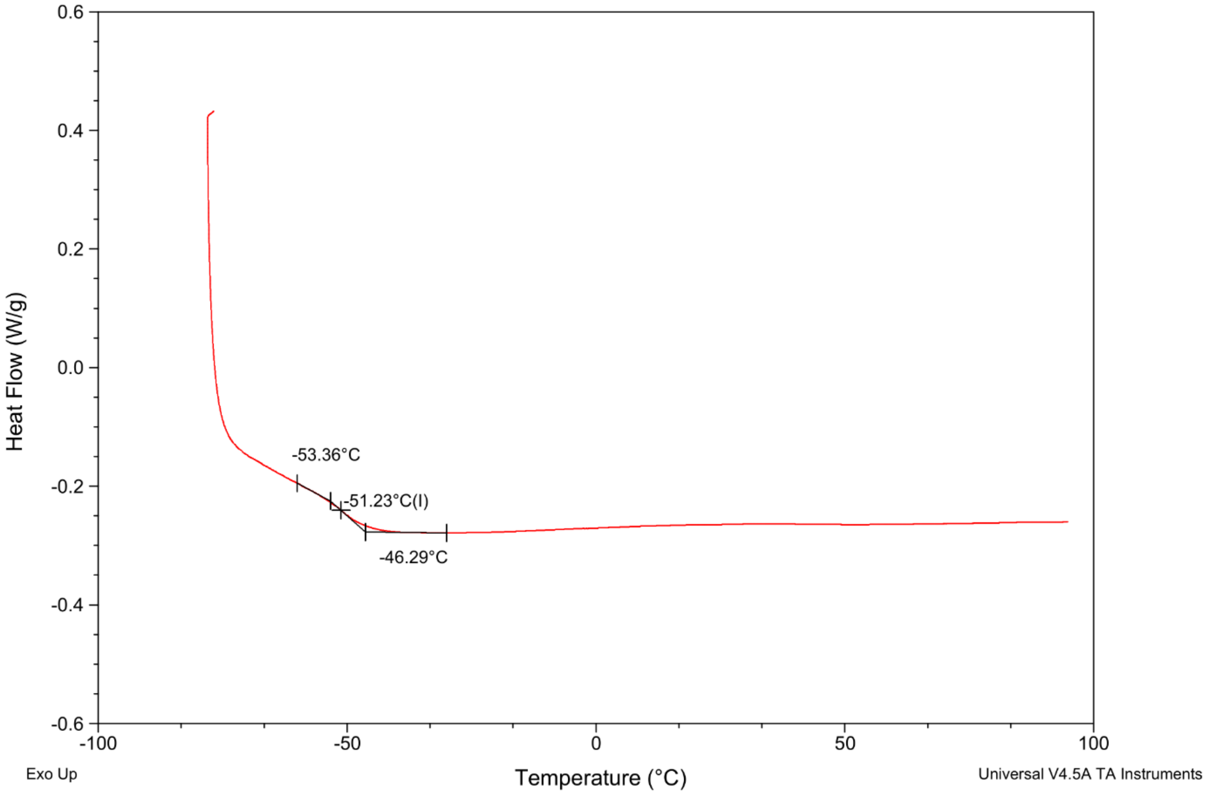

Figure S23. The DSC curve of TP-AspIBU patch.

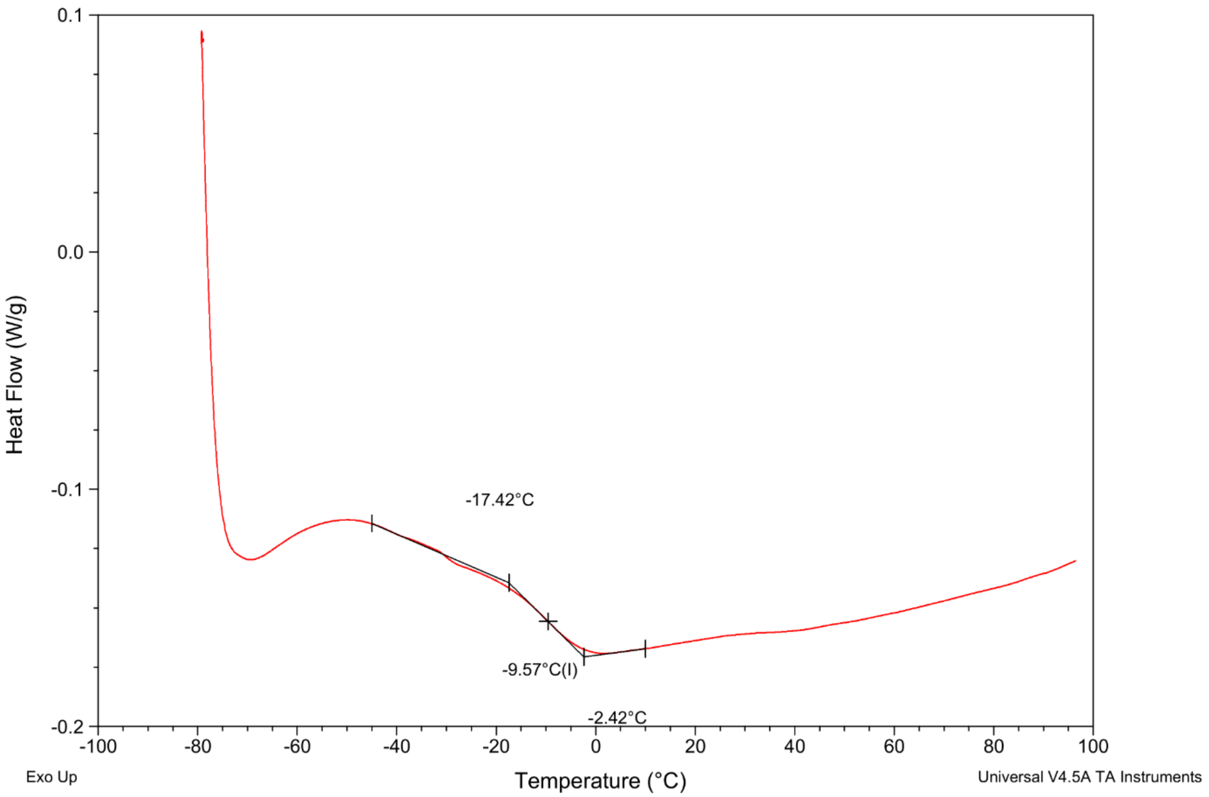

Figure S24. The DSC curve of TP-LysIBU patch.

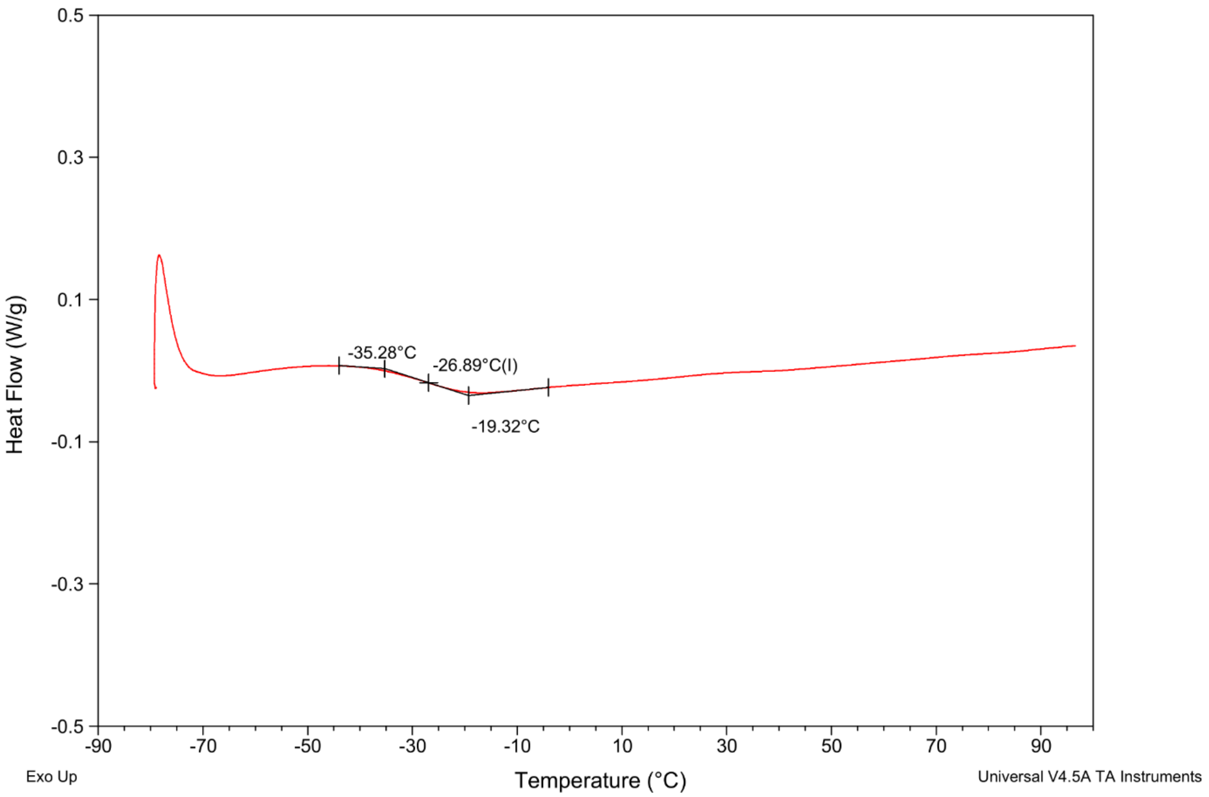

Figure S25. The DSC curve of TP-LysIBU$_2$ patch.

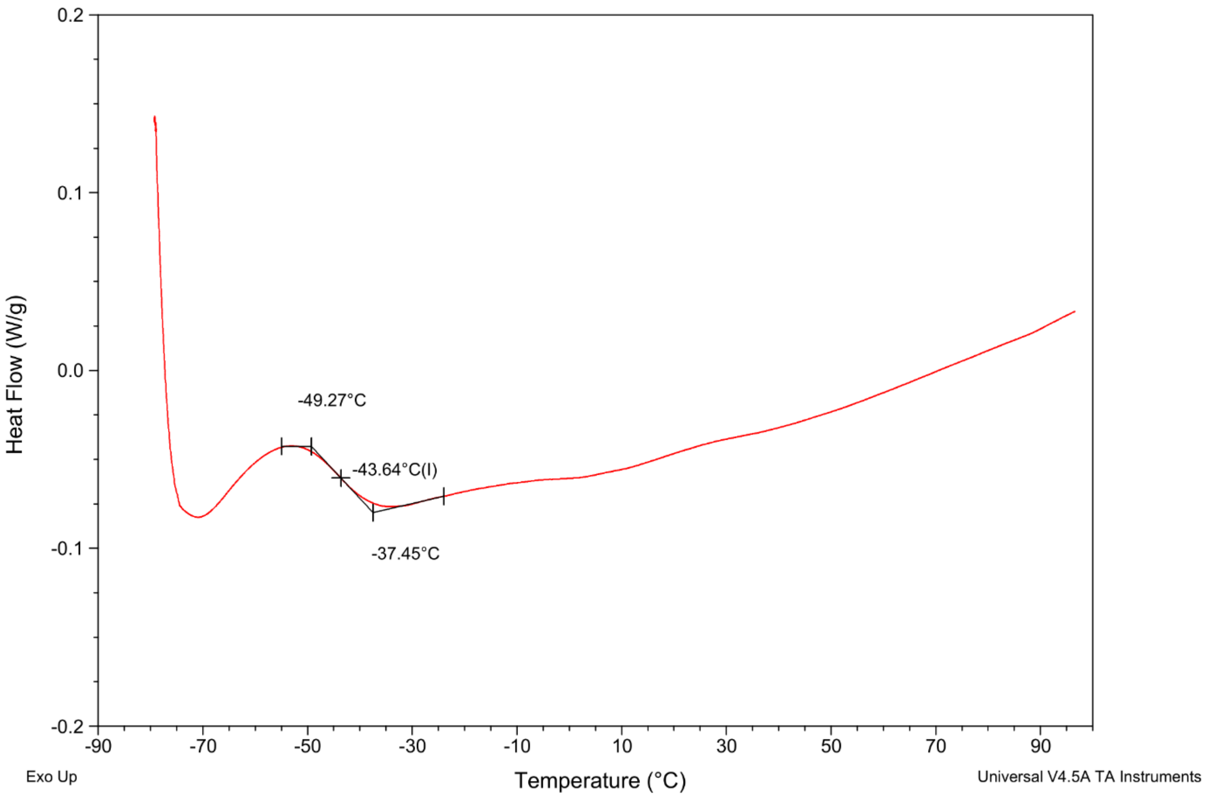

Figure S26. The DSC curve of TP-PheIBU patch.

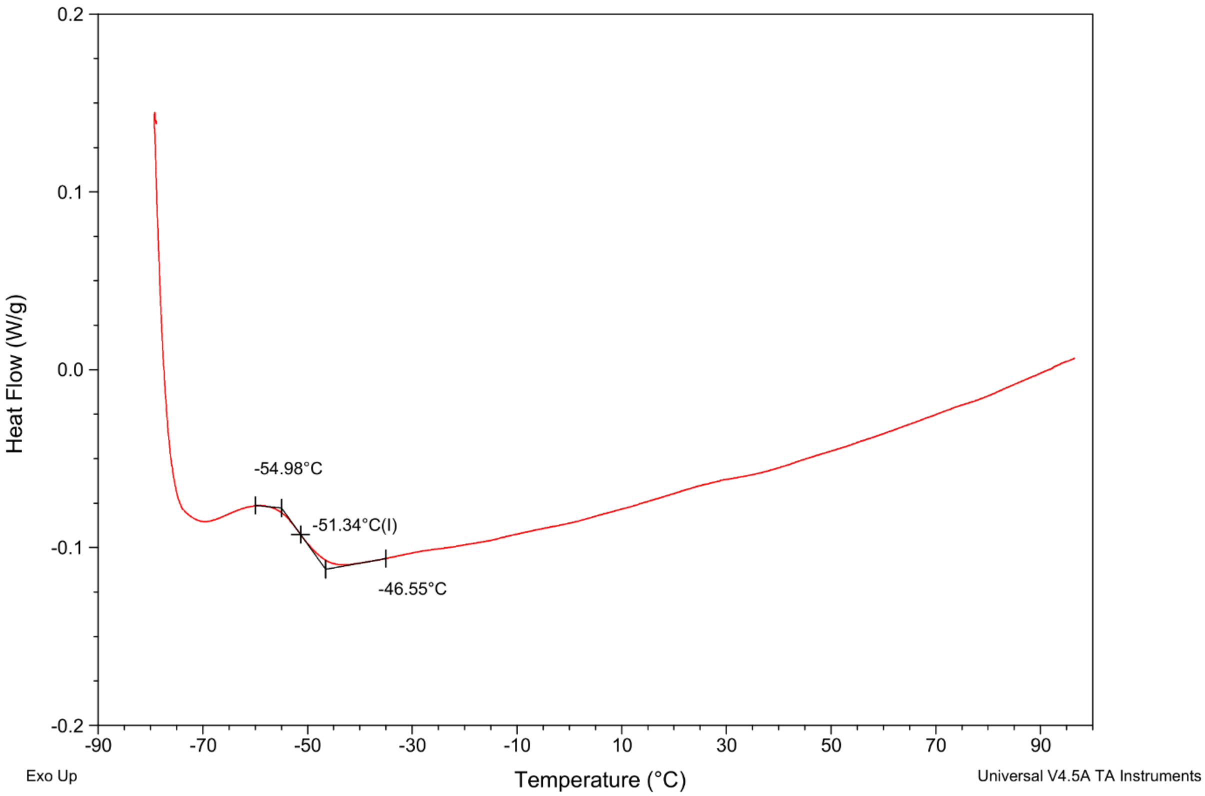

Figure S27. The DSC curve of TP-ProIBU patch.

Table S1. The Mann-Whitney's test estimated significant differences in the cumulative mass between all analyzed compounds, taking all time points during the entire 24 h permeation.

	TP-IBUNa	TP-GlyIBU	TP-AlaIBU	TP-ValIBU	TP-SerIBU	TP-ThrIBU	TP-AspIBU	TP-LysIBU	TP-LysIBU$_2$	TP-PheIBU	TP-ProIBU	CP
TP-IBU	Z = 0.2787<br>p = 2.701	Z = 0.1605<br>*p* = 0.1562	Z = -0.7425<br>*p* = 0.6365	Z = -1.1227<br>*p* = 0.2701	Z = -1.9428<br>*p* = 0.0520	Z = -1.9428<br>*p* = 0.0520	Z = -0.8926<br>*p* = 0.3720	Z = 1.8378<br>*p* = 0.0660	Z = -2.3629<br>*p* = 0.0181*	Z = -1.7328<br>*p* = 0.0831	Z = -0.6826<br>*p* = 0.4948	Z = -0.2625<br>*p* = 0.7928
TP-IBUNa		Z = -1.8378<br>*p* = 0.0660	Z = -1.3127<br>*p* = 0.1892	Z = -1.7328<br>*p* = 0.0831	Z = -2.4679<br>*p* = 0.0135*	Z = -2.2579<br>*p* = 0.0239*	Z = -1.5228<br>*p* = 0.1278	Z = -2.1529<br>*p* = 0.0278*	Z = -2.5730<br>*p* = 0.0100*	Z = -2.3629<br>*p* = 0.0181*	Z = -1.4177<br>*p* = 0.1562	Z = -1.2077<br>*p* = 0.2271
TP-GlyIBU			Z = 0.3675<br>*p* = 0.7131	Z = 0.1575<br>*p* = 0.8748	Z = -1.1027<br>*p* = 0.2701	Z = -1.1027<br>p = 0.2701	Z = 0.4725<br>*p* = 0.6365	Z = -1.2077<br>*p* = 0.2271	Z = -1.7328<br>*p* = 0.0831	Z = -0.5776<br>*p* = 0.5635	Z = 0.6826<br>*p* = 0.4948	Z = 1.1027<br>*p* = 0.2701
TP-AlaIBU				Z = -1.1575<br>*p* = 0.8748	Z = -1.4177<br>*p* = 0.1562	Z = -1.3127<br>*p* = 0.1892	Z = -0.8926<br>*p* = 0.3720	Z = -1.3127<br>*p* = 0.1892	Z = -1.7328<br>*p* = 0.0831	Z = -1.1027<br>*p* = 0.2071	Z = 0.1575<br>*p* = 0.8748	Z = 0.4725<br>*p* = 0.6365
TP-ValIBU					Z = -1.3127<br>*p* = 0.1892	Z = -1.2077<br>*p* = 0.2271	Z = 0.3675<br>*p* = 0.7131	Z = -1.3127<br>*p* = 0.1892	Z = -1.6278<br>*p* = 0.1035	Z = 0.8926<br>*p* = 0.3720	Z = 0.5776<br>*p* = 0.5635	Z = 0.9977<br>*p* = 0.3184
TP-SerIBU						Z = 0.1575<br>*p* = 0.8748	Z = 1.4177<br>*p* = 0.1562	Z = 0.0525<br>*p* = 0.9581	Z = 0.7876<br>*p* = 0.4308	Z = 0.6826<br>*p* = 0.4948	Z = 1.4177<br>*p* = 0.1562	Z = 1.9428<br>*p* = 0.0520
TP-ThrIBU							Z = 1.1212<br>*p* = 0.1892	Z = -0.1575<br>*p* = 0.8748	Z = 0.9977<br>*p* = 0.3184	Z = 0.4725<br>*p* = 0.6365	Z = 1.3127<br>*p* = 0.1892	Z = 1.8378<br>p = 0.0660
TP-AspIBU								Z = -1.4177 *p* = 0.1562	Z = - 1.7328<br>*p* = 0.0831	Z = -1.1027<br>*p* = 0.2071	Z = -0.3675<br>*p* = 0.7131	Z = 0.6826<br>*p* = 0.4948
TP-LysIBU									Z = -0.5776<br>*p* = 0.5635	Z = 0.4725<br>*p* = 0.6365	Z = 1.4117<br>*p* = 0.1562	Z = 1.8378<br>*p* = 0.0660
TP-LysIBU$_2$										Z = 1.2077<br>*p* = 0.2771	Z = 1.9428<br>*p* = 0.0520	Z = 2.2579<br>*p* = 0.0239*
TP-PheIBU											Z = 1.2077<br>*p* = 0.2771	Z = 1.7328<br>*p* = 0.0831
TP-ProIBU												Z = 1.7328<br>*p* = 0.0831
